# Microwave Radiometer Instability Due to Infrequent Calibration

**Published:** 2020

**Authors:** Kevin J. Coakley, Jolene Splett, David Walker, Mustafa Aksoy, Paul Racette

**Affiliations:** National Institute of Standards and Technology, Boulder, CO 80305 USA; National Institute of Standards and Technology, Boulder, CO 80305 USA; National Institute of Standards and Technology, Boulder, CO 80305 USA; University at Albany, State University of New York, Albany, NY 12222 USA; National Aeronautics and Space Administration, Greenbelt, MD 20771 USA

**Keywords:** Calibration, measurement errors, microwave radiometry, random noise, remote sensing, stability criteria, statistics, stochastic processes, uncertainty quantification

## Abstract

We directly quantify the effect of infrequent calibration on the stability of microwave radiometer temperature measurements (where a power measurement for the unknown source is acquired at a fixed time, but calibration data are acquired at variable earlier times) with robust and nonrobust implementations of a new metric. Based on our new metric, we also determine a component of uncertainty in a single measurement due to infrequent calibration effects. We apply our metric to experimental data acquired from experimental ground-based calibration data acquired from a NASA millimeter-wave imaging radiometer and a NIST radiometer (Noise Figure Radiometer-NFRad). Based on a stochastic model for the NFRad, we determine the random uncertainty of an empirical prediction model of our stability metric by a Monte Carlo method. For comparison purposes, we also present a secondary metric that quantifies stability for the case where calibration data are acquired at a fixed time, but power measurements for the unknown source are acquired at variable later times.

## I. Introduction

Microwave radiometers [1], [2] are typically calibrated based on power measurements of reference sources with well-characterized temperatures. Given calibration data acquired from the references, and the measured power of an unknown source, one estimates the temperature of the unknown source. The stability of this estimate depends, in part, on how often one acquires calibration data as well as the particular calibration method employed [3]–[6]. For instance, one might estimate the unknown source temperature at time *t* based on calibration data for two references at some past time *t − τ* or multiple times before and after *t*. In this article, we focus on the first approach that is typically denoted as the “two-point calibration” method.

Existing methods to quantify the stability of radiometer temperature estimates include the variogram [7]–[10] and Allan deviation spectra [11]–[14]. Although valuable, neither approach directly quantifies stability as a function of the variable time delay between the data acquisition time of the calibration data and the time at which the power of the unknown is measured. In this article, we directly quantify how stability depends on this variable time delay and quantify a component of uncertainty due to infrequent calibration. In contrast, neither the variogram nor the Allan deviation provides estimates of the component of uncertainty due to infrequent calibration. To suppress the influence of outliers (e.g., warm-up effects in calibration studies), we construct a robust version of our stability metric. (For a related discussion of robust implementations of the variogram metric and robust statistical analysis in general, see [8] and [15], respectively.) We expect that discrepancies between the robust and nonrobust implementations of any stability metric may facilitate identification of outliers in a radiometer time series. For informational purposes, we also study a second new metric that quantifies instability when calibration data are acquired at a fixed time, but power measurements for the unknown source are acquired at variable subsequent times. We illustrate our metrics with experimental data acquired with the NIST radiometer (Noise Figure Radiometer-NFRad) [16], [17] and ground-based calibration data acquired from NASA’s millimeter-wave imaging radiometer (MIR) [18]. For the NFRad, we attribute discrepancies between robust and nonrobust implementations of our metric to warm-up effects. We exclude data acquired during the “warm-up” period from our analysis. Based on a nonstationary stochastic model for the NFRad instrument, we determine the random uncertainty of our stability metrics by a Monte Carlo method. We expect our methods to apply to other instruments, including the new generation of small satellites [19]–[24], [25].

## II. Methods

### A. Linear Calibration Model

Suppose that the theoretical temperatures and powers of two reference sources are *T*_1_, *T*_2_, *P*_1_, and *P*_2_, and the theoretical temperature and power of an unknown source are *T*_*u*_ and *P*_*u*_, respectively. At time *t*_*c*_, denote the measured powers of the first and second references as ${\tilde{P}}_{1}\left(t_{c}\right)$ and ${\tilde{P}}_{2}\left(t_{c}\right)$, respectively. At time *t*_*u*_, denote the measured power of the unknown source as ${\tilde{P}}_{u}\left(t_{u}\right)$. According to the two-point calibration method (assuming perfect knowledge of *T*_1_ and *T*_2_), we estimate the temperature of the unknown source at time *t*_*u*_ as ${\tilde{T}}_{u}\left(t_{u}\right)$, where

(1) $${\tilde{T}}_{u}\left(t_{u}\right)=T_{1}+\left[\frac{{\tilde{P}}_{u}\left(t_{u}\right)-{\tilde{P}}_{1}\left(t_{c}\right)}{{\tilde{P}}_{2}\left(t_{c}\right)-{\tilde{P}}_{1}\left(t_{c}\right)}\right]\left(T_{2}-T_{1}\right).$$

For compactness, we write ${\tilde{T}}_{u}\left(t_{u}\right)$ as

(2) $${\tilde{T}}_{u}\left(t_{u}\right)=\tilde{T}\left({\tilde{\theta }}_{t_{c}},{\tilde{P}}_{u}\left(t_{u}\right)\right)$$

where a calibration dataset acquired at time *t*_*c*_ is ${\tilde{\theta }}_{t_{c}}=\left({\tilde{P}}_{1}\left(t_{c}\right),{\tilde{P}}_{2}\left(t_{c}\right)\right)$. For the special case where power measurements are free of random error but systematic error varies linearly with time, the bias (systematic error) of the temperature estimate for the unknown source is

(3) $${\tilde{T}}_{u}-T_{u}=\left[\frac{\beta \left(t_{u}-t_{c}\right)}{P_{2}\left(t_{c}\right)-P_{1}\left(t_{c}\right)}\right]\left(T_{2}-T_{1}\right)$$

where *β* is the temporal derivative of the systematic error for any power measurement.

Here, we focus on quantification of instability due to stochastic effects. For the experimental cases studied here, the primary source of instability in radiometer temperature estimates is random electronic gain variation with a complicated correlation structure. In general, if there are deterministic trends in an observed radiometer time series, one should ideally detrend the time series and determine stability metrics from the residual time series. As a caveat, it may be difficult in some applications to distinguish deterministic trends from stochastic variations.

### B. Stability Metrics

In our primary analysis method, the power of the unknown is measured at some fixed time *t*, but the calibration data are acquired at each of many variable times *t − τ*, where *τ* is nonnegative. We define a “variable calibration (VC)” deviation as *ϵ*_VC_(*t, τ* ), where

(4) $$\epsilon_{\text{VC}}(t,\tau )=\tilde{T}\left({\tilde{\theta }}_{t},{\tilde{P}}_{u}(t)\right)-\tilde{T}\left({\tilde{\theta }}_{t-\tau },{\tilde{P}}_{u}(t)\right).$$

In a secondary analysis, the calibration data are acquired at some fixed time *t*, but power of the unknown is acquired at each of many variable times *t* + *τ*, where *τ* is nonnegative. We define a “fixed calibration (FC)” deviation, *ϵ*_FC_(*t, τ* ), as

(5) $$\epsilon_{\text{FC}}(t,\tau )=\tilde{T}\left({\tilde{\theta }}_{t},{\tilde{P}}_{u}(t+\tau )\right)-\tilde{T}\left({\tilde{\theta }}_{t},{\tilde{P}}_{u}(t)\right).$$

In our analysis, we assume that the observed deviation *ϵ*_VC_(*t, τ*) is a realization of a random variable that has an expected value of 0 and finite second moment (expected squared value) < $\epsilon_{\text{VC}}^{2}(t,\tau ))$ > that does not vary with *t*. The theoretical value of our primary stability metric for quantifying the effect of infrequent calibration is

(6) $$\mathrm{SVC}(\tau )=\sqrt{\frac{1}{2}<\epsilon_{\text{VC}}^{2}(t,\tau )>}$$

where < *X* > denotes the expected value of a random variable *X* observed in statistically similar experiments. We estimate the theoretical value of SVC(*τ*) as

(7) $$\tilde{\mathrm{SVC}}(\tau )=\sqrt{\frac{1}{2}\bar{\epsilon_{\text{VC}}^{2}(t,\tau )}}$$

where $\bar{\epsilon_{\text{VC}}^{2}(t,\tau )}$ is the sample mean (determined from experimental data) of squared VC deviations corresponding to all distinct values of (*t − τ, t*) where data are acquired. The robust version of our (7) estimate is

(8) $$\tilde{\mathrm{RSVC}}(\tau )=\frac{1.4826}{\sqrt{2}}\mathrm{MAD}\left(\epsilon_{\text{VC}}(t,\tau )\right)$$

where MAD(*ϵ*_VC_(*t, τ*)) is the median absolute deviation (MAD) of the set of *ϵ*_VC_(*t, τ*) deviations corresponding to all distinct values of (*t − τ, t*), where data are acquired. To get the MAD of *n* values, (*x*_1_, *x*_2_, …, *x*_*n*_), one first computes their median *x*_med_. The MAD is the median of the following absolute deviations (*|x*_1_ *− x*_med_*|, |x*_2_ *− x*_med_*|,…, |x*_*n*_ *− x*_med_*|*). As discussed in many references, including [26], the factor 1.4826 in (8) ensures that the scaled MAD of many realizations of a Gaussian random variable converges to the standard deviation of the Gaussian distribution (to within five digit precision). This follows from the observation that when applied to random variables with symmetric distributions, the MAD converges to 1/2 times the interquartile range of the distribution [15], which is approximately 0.67448 *σ* for a Gaussian distribution with standard deviation *σ*.

For cases where the asymptotic limit of $\tilde{\mathrm{RSVC}}(\tau )$ determined from data pooled from *N* statistically similar and independent experimental datasets as *N →∞* is defined, the theoretical value of RSVC is this limit. We define theoretical values for the nonrobust and robust version of our second metric in a similar way for $\tilde{\mathrm{SFC}}(\tau )$ and $\tilde{\mathrm{RSFC}}(\tau )$ in terms of the deviation *ϵ*_FC_(*t, τ*).

## III. Results

### A. NIST Radiometer

#### 1) NFRad Measurement System

At the NIST, noise powers from sources are measured with NFRad—a total-power noise radiometer [16], [17]. The NFRad does not detect power with a typical square-law detector (such as diodes and thermocouples). Instead, the NFRad detects the power of a source with a thermistor that responds to injected RF power by adjusting the amount of dc power dissipated in the thermistor. The RF power of the source is inferred from the reduction of dc power necessary to maintain the thermistor in its original state (where no RF power is injected) based on the dc substitution principle [27].

The NFRad consists of an ultralinear amplifier chain terminated with a NIST Type-IV power meter. The ultralinear amplifier chain refers to the entire radiometer detection, including the amplifier, mixer, and detector. The two calibration noise standards are an ambient 50-Ω load and a cryogenic load immersed in a liquid nitrogen bath. Multiple noise sources may be connected to the measurement system at any one time to facilitate intercomparisons between devices.

We estimate the power of any source (reference or unknown) as $\tilde{P}$, where

(9) $$\tilde{P}=\frac{{\tilde{V}}_{\text{off}}^{2}-{\tilde{V}}_{\text{source}}^{2}}{\tilde{R}}$$

where ${\tilde{V}}_{\text{off}}$ corresponds to measured voltage when no RF power is injected, ${\tilde{V}}_{\text{source}}$ corresponds to measured voltage when RF power from a source is injected, and $\tilde{R}=200\;\Omega$ is the nominal resistance of the thermistor in the power meter. The uncertainty of this resistance is negligible.

The NFRad operates in a band between 1 and 12 GHz. During each cycle of the experiment, we measure four voltages: *V*_off_ and *V*_source_ (for the unknown source and the two calibration sources). For each case, the average of ten repeat voltage measurements (acquired every 50 ms) are averaged. The wait time between the different cases within any cycle is 500 ms. For analysis purposes, we assume that the data acquisition times for power measurements within any cycle are the same. The interval between cycles is approximately 26 s.

#### 2) Experimental Data

**Fig. 1.**
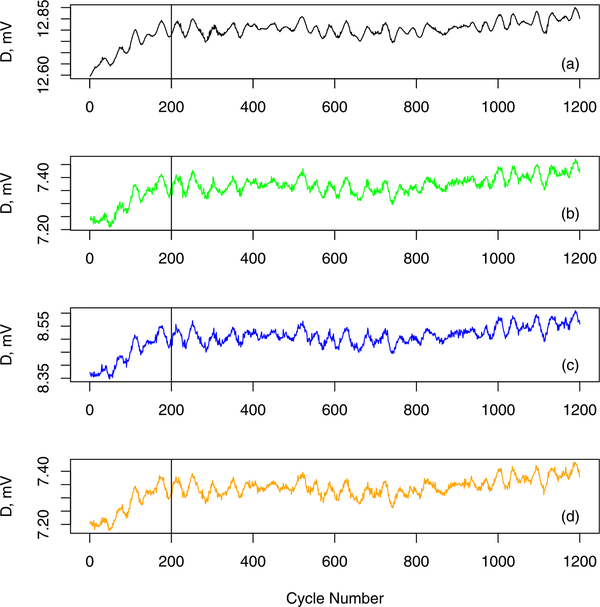
Observed NFRad time series for (a) no source, (b) ambient source, (c) cryogenic source, and (d) warm source. Solid vertical line is the data selection threshold. To improve the resolution of the *y*-axis scale, we plot the difference, *D*, between the observed voltage and 2.62 V. For each cycle, we assume that powers are determined for the unknown and reference sources simultaneously. The time interval between successive cycles is approximately 26 s.

With the NFRad, we observe voltage time series corresponding to three sources labeled as “Warm,” “Ambient,” and “Cryogenic” (see Fig. 1). The corresponding temperatures for the three sources are 302.9, 296.9, and 84.25 K. In our study, the “Warm” source serves as the unknown source, and the other two sources serve as calibration reference sources.

**Fig. 2.**
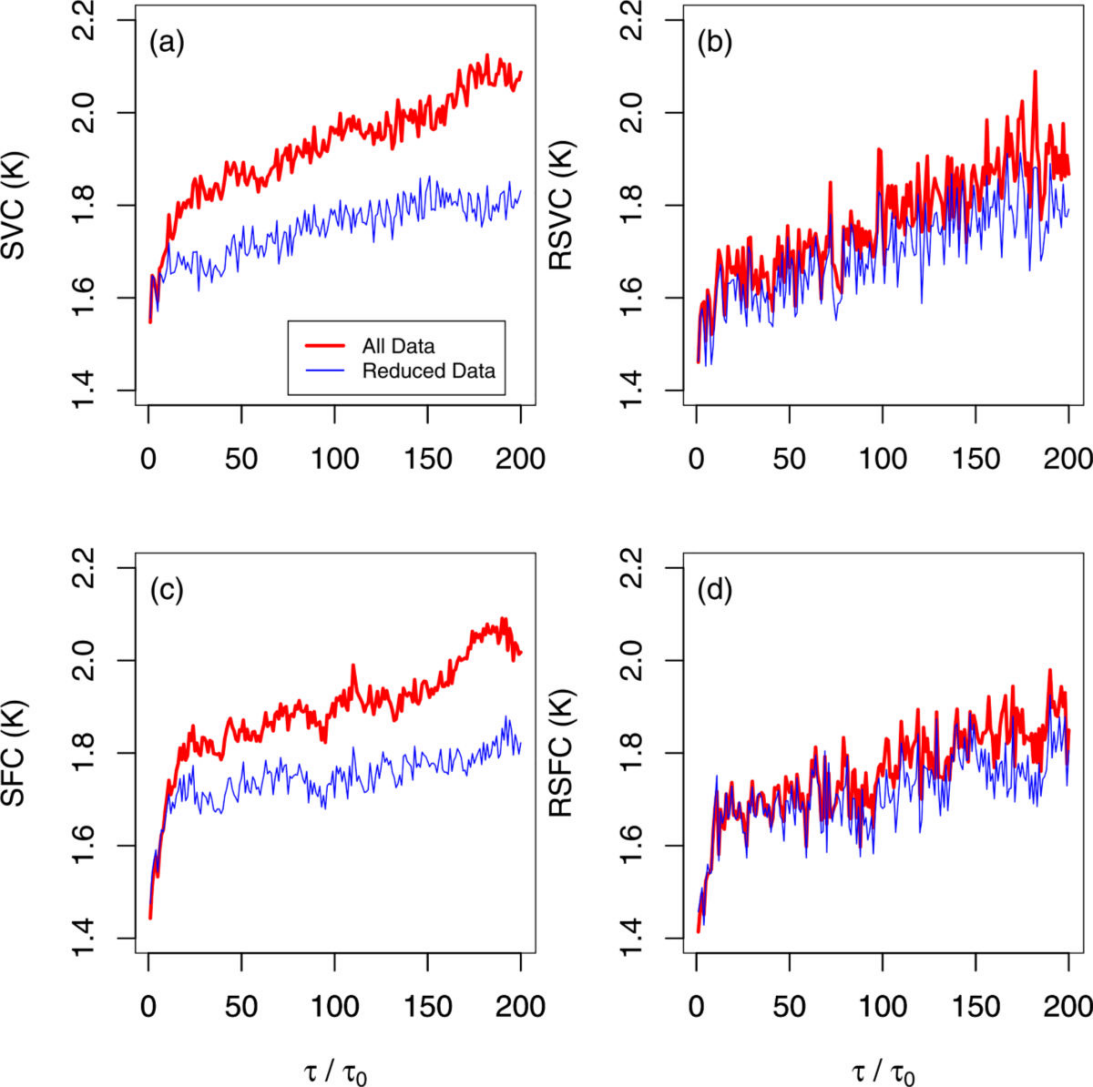
Metrics determined from NFRad data. (a) Estimated SVC from full and reduced data. The reduced data excludes the first 200 cycles of data (see Fig. 1). (b) Estimated RSVC from same data as analyzed in (a). (c) Estimated SFC from full and reduced data. (d) Estimated RSFC from same data as analyzed in (c). The interval between adjacent cycles (where data are acquired) is *τ*_*o*_ *≈* 26 s. The normalized lag, *τ/τ*_*o*_, takes positive integer values (1, 2, 3,…).

In NFRad experiments, early measurements are typically unreliable due to warm-up effects. When we determine our stability metrics from the full data (including the first 200 cycles), SVC and RSVC are not in good agreement [see Fig. 2(a)].We expect this discrepancy since SVC does not down-weight outliers due to warm-up effects, whereas RSVC does. In contrast, when we determine these metrics from the reduced data (which excludes the first 200 cycles), the two metrics are in good agreement [see Fig. 2(b)]. Thus, the dramatic discrepancy between SVC and RSVC (when determined from the full data) indicates the presence of outliers due to warm-up effects. In general, we expect that comparison studies of SVC and RSVC may serve as a diagnostic for detecting other sorts of outliers besides those produced by warm-up effects. Similar comments apply to SFC and RSFC [see Fig. 2(c) and (d)].

#### 3) Observed Stability Metrics

**Fig. 3.**
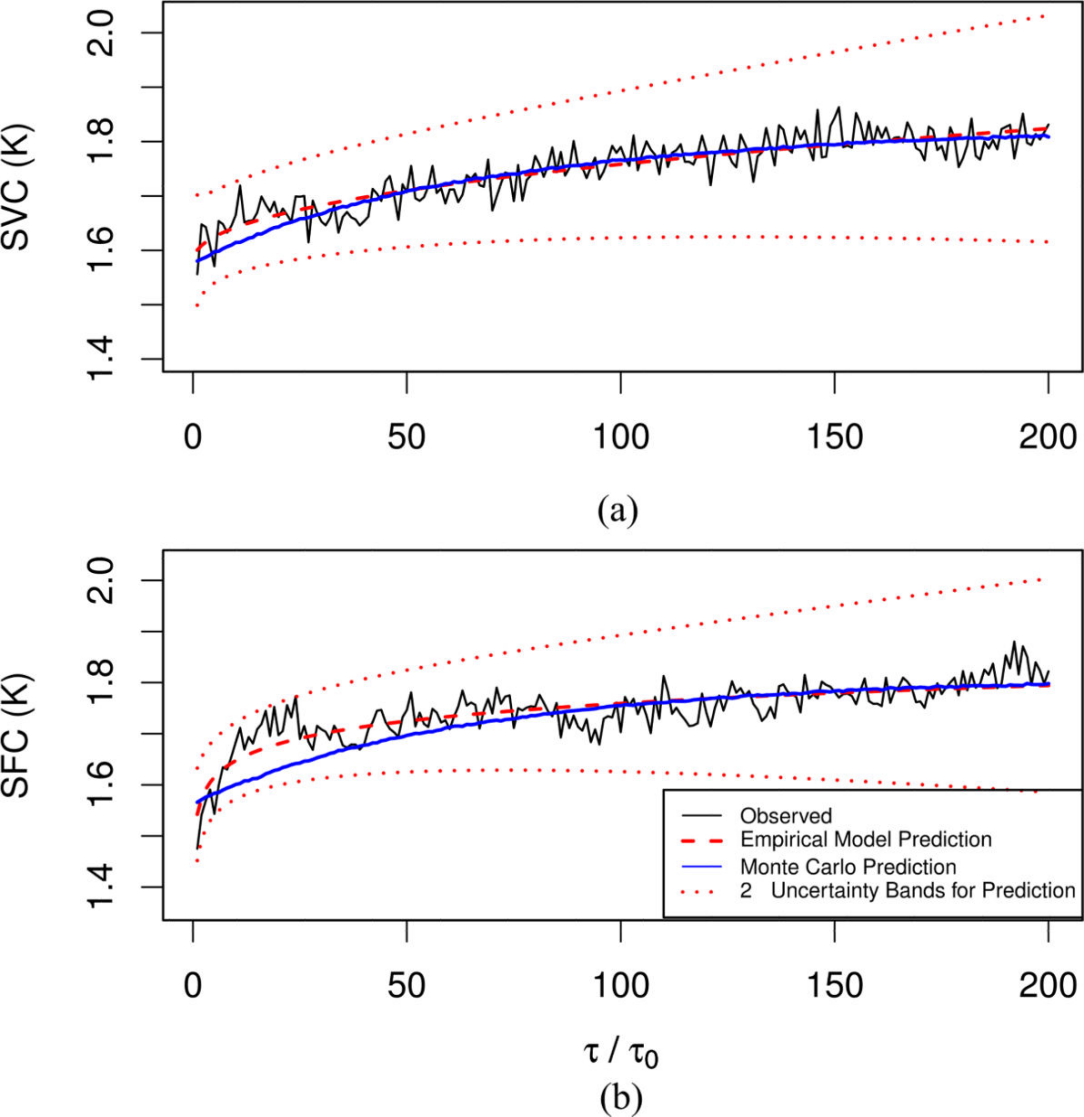
Stability metrics determined from reduced NFRad data. (a) Estimated SVC. (b) Estimated SFC. We predict the metrics determined from observed data with empirical models [see (10) and (11)]. We show the average of metrics determined from 500 realizations of simulated data (see the Appendix). We denote this average as the Monte Carlo prediction. The relative uncertainties of the predicted metric values determined by our empirical models [see (10) and (11)] generally increase with lag and range from approximately 2.5–5.5% for the lags shown. The interval between adjacent cycles (where data are acquired) is *τ*_0_ *≈* 26 s. The normalized lag, *τ/τ*_0_, takes positive integer values (1, 2, 3,…).

We determine our metrics from the reduced data, which correspond to a time series with 1000 samples. The spacing between samples is *τ*_0_ = 26s. Hence, the time series that we analyze was acquired during an observation time of 7.2 h. For *τ /τ*_*o*_ *≥* 1 (where *τ*_0_ *≈* 26 s), we characterize observed values of SVC and SFC (see Fig. 3) with the following empirical prediction models:

(10) $$\hat{\mathrm{SVC}}\left(\tau /\tau_{o}\right)=\left(\alpha_{1}+\beta_{1}{\left(\tau /\tau_{o}\right)}^{\gamma_{1}}\right)\text{K}$$

and

(11) $$\hat{\mathrm{SFC}}\left(\tau /\tau_{o}\right)=\left(\alpha_{2}+\beta_{2}{\left(\tau /\tau_{o}\right)}^{\gamma_{2}}\right)\text{K}.$$

We determine the model parameters by the method of least squares, where all parameter estimates are constrained to be nonnegative (see Table I).

**Fig. 4.**
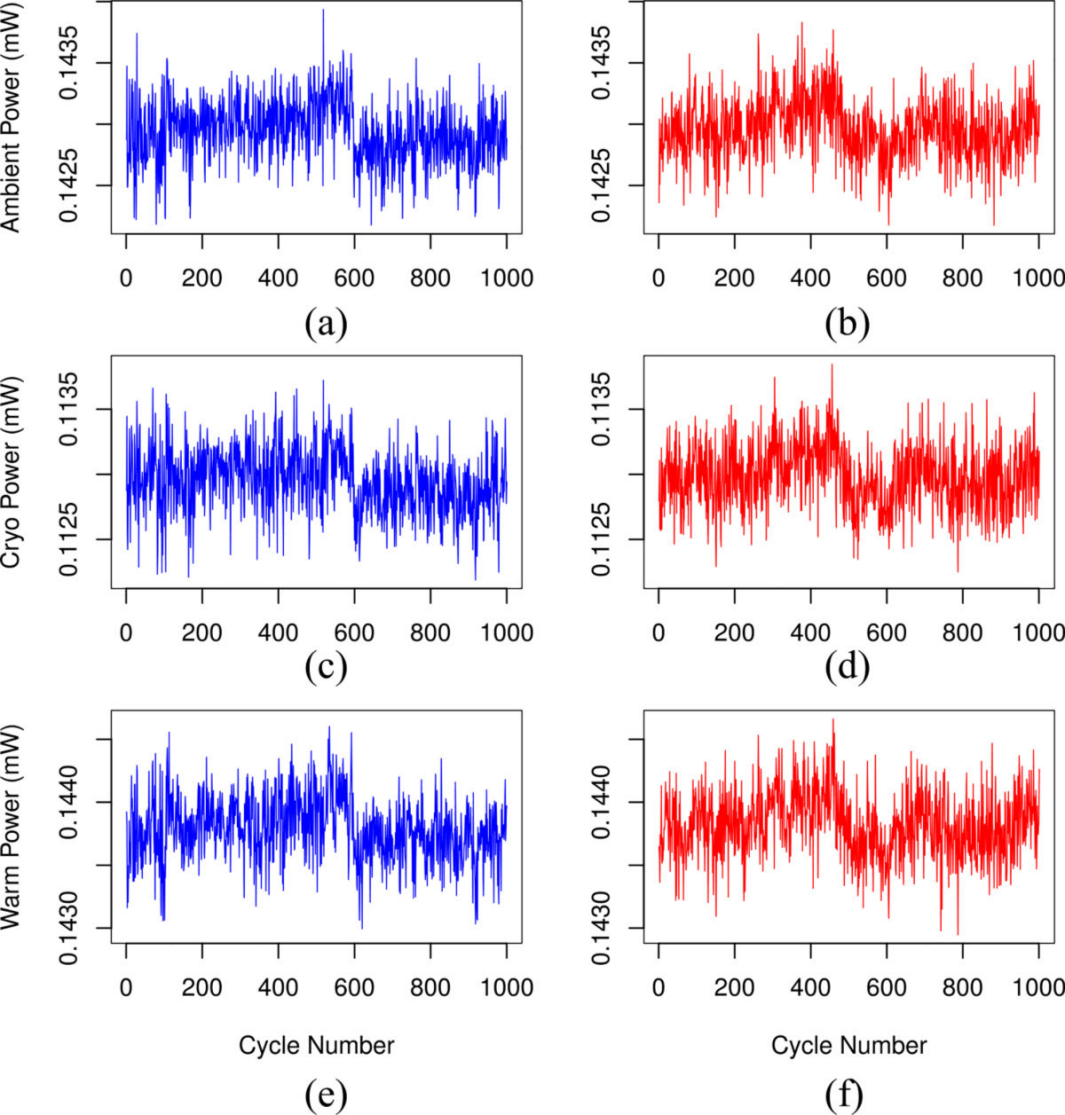
NFRad power time series for (a) observed ambient power, (b) simulated ambient power, (c) observed cryogenic power, (d) simulated cryogenic power, (e) observed warm power, and (f) simulated warm power. For each cycle, we assume that powers are determined for the unknown and reference sources simultaneously. The interval between adjacent cycles (where data are acquired) is *τ*_0_ *≈* 26 s.

We model each observed NFRad voltage time series as a realization of a nonstationary stochastic processes (see the Appendix). Based on the values of the simulation model parameters determined from the observed NFRad data, we simulate many realizations of the voltage time series. From each realization of a set of four simulated voltage time series (*V*_off_, unknown source voltage, and voltages for the two calibration sources), we determine power time series for the unknown and the two calibration reference sources in exactly the same way that we determined power time series from the observed NFRad data. (See Fig. 4 for comparison of observed and example realizations of simulated power.)

From each realization of a set of simulated power time series, we compute SVC and SFC metrics at all lags of interest and determine the prediction model [see (10) and (11)] parameters. From these results, we determine the standard errors of the prediction model [see (10) and (11)] parameters (see Table I) and the standard error of the prediction at each lag (see Fig. 3). Even though the relative uncertainties of the model parameters shown in Table I are generally greater than 50%, the lag-dependent relative uncertainties of the empirical prediction models [see (10) and (11)] range from 2.5% to 5.5% (see Fig. 3).

### B. NASA Radiometer

#### 1) Experimental Data

The NASA MIR is a five-receiver airborne radiometer built for remote sensing of water vapor, precipitation, and clouds [18]. The MIR is a total power radiometer with periodic through-the-antenna calibrations with two internally mounted blackbody references. We analyze data acquired from a 6-h laboratory experiment configuration of MIR (see [28] for more details). Three calibration targets were viewed periodically. The integration time for each measurement was 200 ms. Accounting for latency between target views, the cycling time was 1.16 s. Based on measured powers for the references and unknown, temperature is estimated with (1). The temperatures of the hot and cold reference sources are approximately 325.6 and 293.7 K, respectively. A third source with a temperature of approximately 79.0 K serves as the unknown source in our study.

### C. Stability Metrics

**Fig. 5.**
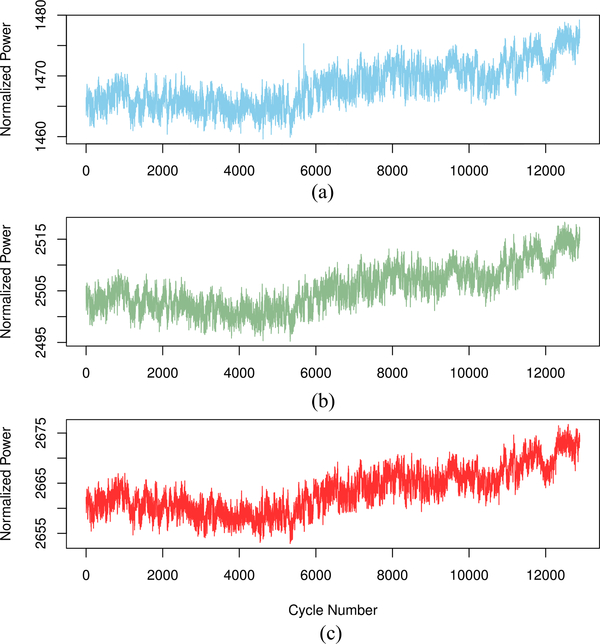
NASA MIR power time series. (a) Unknown source. (b) Cold reference source. (c) Hot reference source. The interval between adjacent cycles where data are acquired is approximately 1.16 s.

**Fig. 6.**
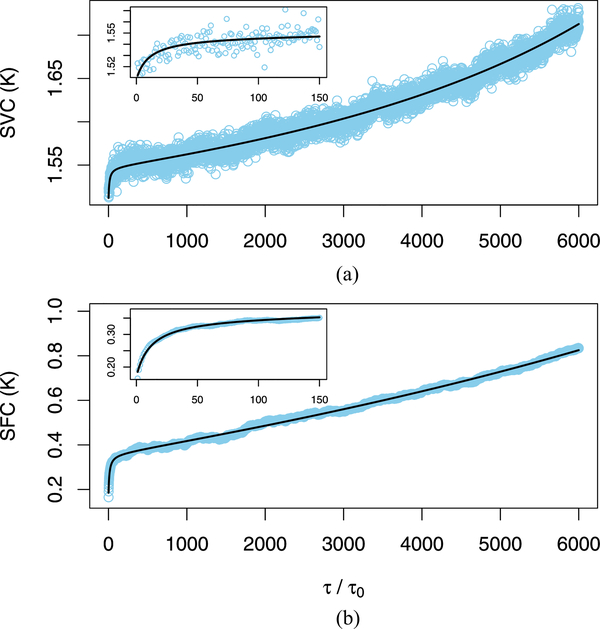
Stability metrics determined from MIR data. In (a) and (b), we predict the observed metrics defined in (12) and (13) with rational function models. The interval between adjacent cycles (where data are acquired) is *τ*_*o*_ *≈* 1.16 s. The normalized lag, *τ/τ*_*o*_, takes positive integer values (1, 2, 3,…).

As a safeguard against possible warm-up effects, we fit the above models to a subset of the calibration data that excludes the first 28% of the calibration data. The number of samples in the time series that we determine our metrics from is 12 890. We stress that we analyze only a subset of all the data presented in [28]. The spacing between samples is *τ*_0_ *≈* 1.16 s. Hence, the time series that we analyze (see Fig. 5) corresponds to a data acquisition time of 4.15 h. We characterize the observed values of SVC and SFC with empirical prediction models determined with model fitting software [29] (see Fig. 6 and Table II) as

(12) $$\hat{\mathrm{SVC}}\left(\tau /\tau_{0}\right)=\frac{a_{1}+c_{1}\left(\tau /\tau_{o}\right)+e_{1}{\left(\tau /\tau_{o}\right)}^{2}}{1+b_{1}\left(\tau /\tau_{o}\right)+d_{1}{\left(\tau /\tau_{o}\right)}^{2}}\text{K}$$

and

(13) $$\hat{\mathrm{SFC}}\left(\tau /\tau_{0}\right)=\frac{a_{2}+c_{2}\left(\tau /\tau_{o}\right)+e_{2}{\left(\tau /\tau_{o}\right)}^{2}}{1+b_{2}\left(\tau /\tau_{o}\right)+d_{2}{\left(\tau /\tau_{o}\right)}^{2}}K.$$

In contrast to the NFRad analysis, we failed to develop a stochastic model for the MIR data and determine standard errors for metric model parameters by Monte Carlo methods. Identification of such a stochastic model for the MIR data is a worthy topic but beyond the scope of this article.

For the NFRad, the maximum (minimum) values of observed SVC and SFC [see Fig. 3(a) and (b)] are similar for the lags considered. However, for the MIR, the maximum value (over all lags) of observed SVC (about 1.7 K) is over 50% larger than the maximum value of observed SFC (about 0.8 K). Furthermore, the minimum value of observed SVC (about 1.5 K) is much larger than the minimum value of observed SFC (about 0.2 K). This disagreement is very plausible because the difference of the temperatures of two MIR reference sources (31.90 K) is much less than than the difference of the temperatures of the two NFRad reference sources (212.65 K). Hence, for the MIR, it is very plausible that the denominator term in (1) produces more variability in SVC than in SFC.

## IV. Remarks

### A. Uncertainty Component Due to Infrequent Calibration

For any value of *τ >* 0, no matter how small, we expect SVC(*τ* ) to be nonzero, since random errors in power measurements for any two distinct times are different. Hence, SVC has a discontinuity at *τ* = 0. In the variogram literature, a similar effect called the nugget effect can produce a discontinuity in the variogram at lag 0. (See [7] for more discussion of this point.) For the two radiometers, the empirical prediction model parameters *α*_1_ in (10) and *a*_1_ in (12) correspond to the discontinuity in SVC/K at *τ* = 0. Assuming that the expected value of the deviation defined in (4) is 0, 2SVC^2^(*τ*) is a variance. We decompose this variance into the sum of an irreducible variance $u_{\text{ir}}^{2}$ and a variance associated with infrequent calibration effects $u_{\text{ic}}^{2}(\tau )$ (e.g., temporal variations in gain). Assuming that measurement errors produced by these two effects are independent, we conclude that

(14) $$2{\mathrm{SVC}}^{2}(\tau )=u_{\text{ir}}^{2}+u_{\text{ic}}^{2}(\tau )$$

where the irreducible variances for the NFRad and the MIR are (*u*_ir_*/*K)^2^ = 2(*α*_1_)^2^ and (*u*_ir_*/*K)^2^ = 2(*a*_1_)^2^, respectively. Given an estimate of *u*_ir_, ${\tilde{u}}_{\text{ir}}$, we quantify the component of uncertainty due to infrequent calibration in any one temperature measurement (given that $2{\hat{\text{SVC}}}^{2}(\tau )\geq {\tilde{u}}_{\text{ir}}^{2}$) as

(15) $${\tilde{u}}_{\text{ic}}(\tau )=\sqrt{2{\hat{\text{SVC}}}^{2}(\tau )-{\tilde{u}}_{\text{ir}}^{2}}.$$

For other cases, ${\tilde{u}}_{\text{ic}}(\tau )=0$.

**Fig. 7.**
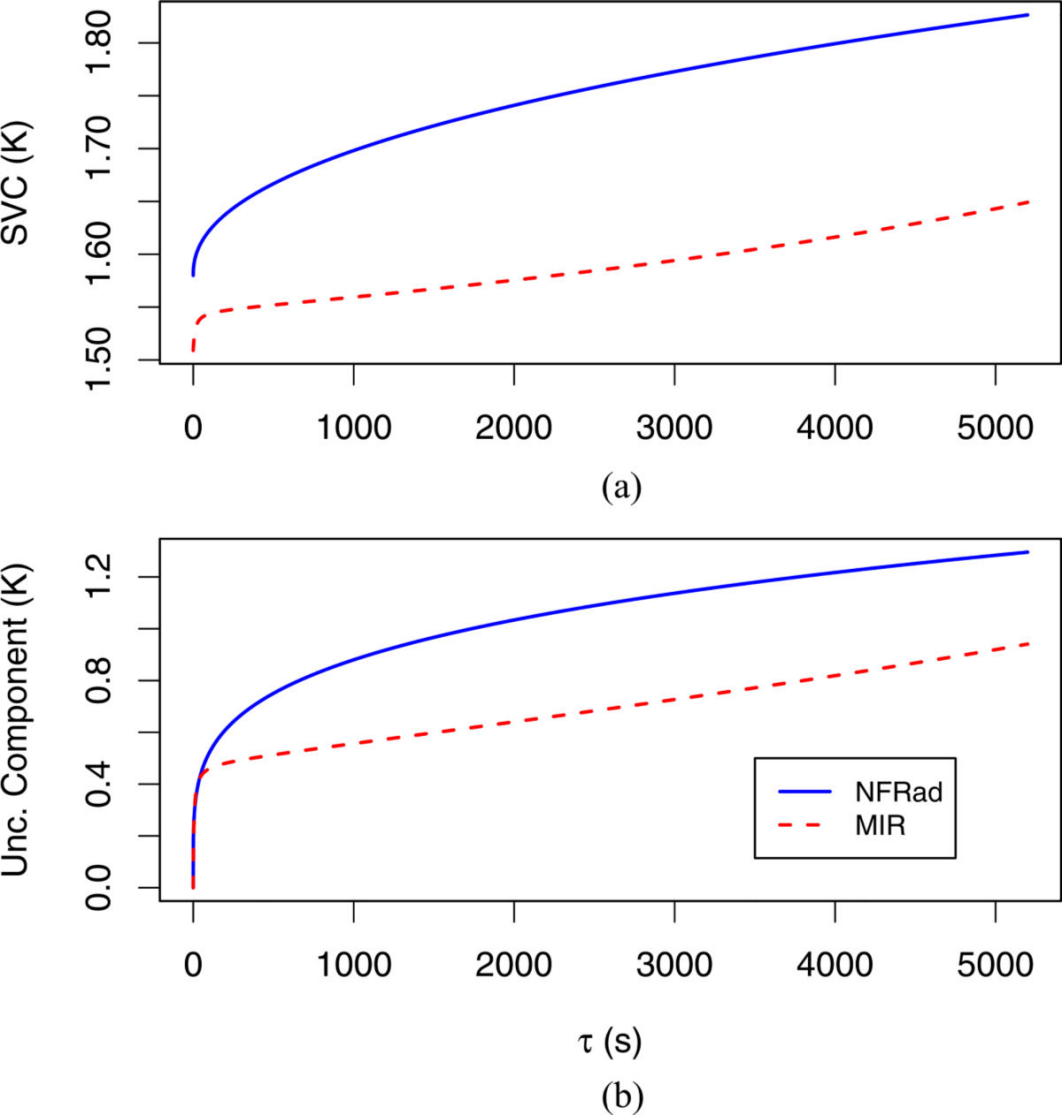
(a) Empirical prediction models for SVC for the NFRad [see (10)] and the MIR [see (12)]. (b) Estimate of component of uncertainty due to infrequent calibration, ${\tilde{u}}_{\text{ic}}(\tau )$ (15), for a measurement of the temperature of the unknown source.

For the NFRad and the MIR, the estimates of *α*_1_ and *a*_1_ (listed in Tables I and II) are 1.58 and 1.51, respectively. In Fig. 7, we show how ${\tilde{u}}_{\text{ic}}(\tau )$ varies with *τ* for both radiometers. We stress that neither the variogram metric nor the Allan variance metric yields an estimate ${\tilde{u}}_{\text{ic}}(\tau )$.

For the case where the power of the unknown at a particular cycle is determined with calibration data acquired at the same cycle, i.e., for the case where *τ* = 0, we denote the theoretical variance of the temperature estimate at each time as $u_{c}^{2}$ (assuming that the observed temperature time series is stationary). For the general case where calibration data and the unknown are not acquired during the same cycle, we express the theoretical variance of the temperature estimate as *u*^2^_combined_(*τ*), where

(16) $${u^{2}}_{\mathrm{combined}}(\tau )=u_{c}^{2}+u_{\text{ic}}^{2}(\tau ).$$

We determine $u_{c}^{2}$ based on the analysis of the observed time series of temperature estimates, where powers of the unknown and calibration reference sources are acquired at the same cycle. In particular, with the auto.arima function [30] in R [31] (a public domain software system), we fit various candidate autoregressive-integrated moving average (ARIMA) models (see the Appendix for a definition of ARIMA models) to the temperature time series, and select the one that minimizes the corrected Akaike information criterion (AICc) [32], [33]. For the NFRad and the MIR, the selected models are AR(1) and AR(5), respectively. Since AR models are stationary, our model selection results are consistent with the hypothesis that the observed time series for the *τ* = 0 case is stationary. The associated estimates of *u*_*c*_ and ${\tilde{u}}_{c}$, for the NFRad and the MIR are 1.79 and 1.49 K, respectively. For the case where *τ >* 0, we express the combined uncertainty of the temperature estimate as

(17) $${\tilde{u}}_{\text{combined}}(\tau )=\sqrt{{\tilde{u}}_{c}^{2}+{\tilde{u}}_{\text{ic}}^{2}(\tau )}.$$

In summary, the major steps in our analysis for general applications are the following.

1. For each *τ >* 0, estimate SVC(*τ* ) from all the data [see (4) and (7)].
2. Estimate the discontinuity in SVC at *τ* = 0 with an appropriate empirical model for SVC(*τ*).
3. Estimate the irreducible variance $u_{\text{ir}}^{2}$ as twice the square of the discontinuity determined in step 2.
4. Determine the component of uncertainty due to infrequent calibration at lag *τ* as: ${\tilde{u}}_{\text{ic}}(\tau )=\sqrt{2{\hat{\text{SVC}}}^{2}(\tau )-{\tilde{u}}_{\text{ir}}^{2}}$, where $\hat{\mathrm{SVC}}(\tau )$ and ${\tilde{u}}_{\text{ir}}^{2}$ are estimates of SVC(*τ*) and the irreducible variance $u_{\text{ir}}^{2}$, respectively.

### B. Comparison of Stability Metrics

For the MIR data, we compare variograms to our SVC metric (shown previously in Fig. 6). We define the variogram at lag *τ* as

(18) $$\gamma (\tau )=<\epsilon_{\text{var}}^{2}(t,\tau )>$$

where

(19) $$\epsilon_{\text{var}}(t,\tau )=\tilde{T}\left({\tilde{\theta }}_{t},{\tilde{P}}_{u}(t)\right)-\tilde{T}\left({\tilde{\theta }}_{t-\tau },{\tilde{P}}_{u}(t-\tau )\right).$$

**Fig. 8.**
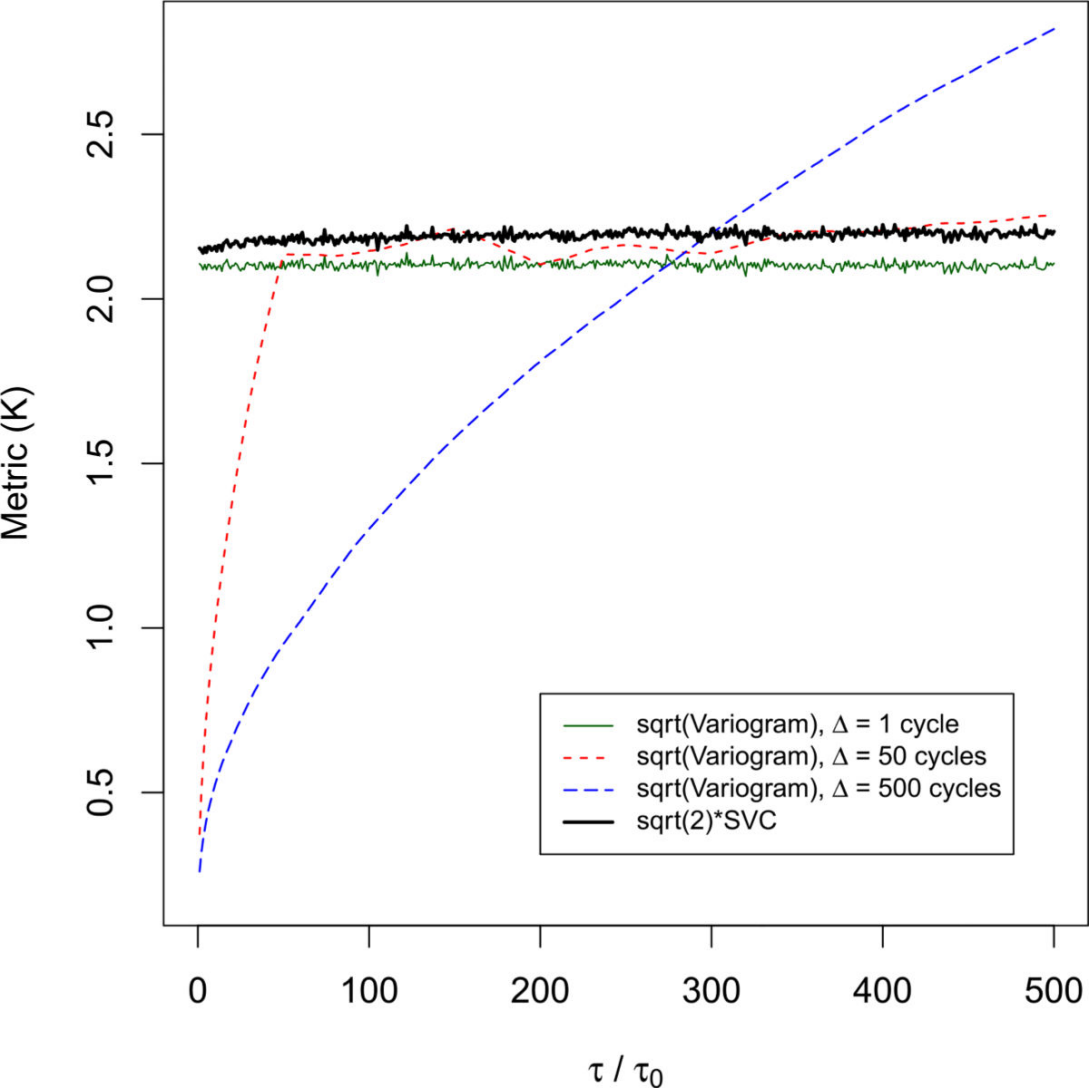
For MIR data, we compare $\tilde{\text{SVC}}$ (same as in Fig. 6) to variograms determined for cases where the interval between acquisition of calibration data, Δ, varies. As described in Section IV-B, variograms for Δ *>* 1 cycle correspond to the case where earlier (relative to times when the power of the unknown source is determined) calibration data are assumed for power estimation. For Δ=1 cycle, at each cycle, power measurements of the unknown source are determined with calibration data acquired at that cycle.

In the variogram study, temperature estimates are determined at each cycle. However, distinct calibration data for the calibration sources are not acquired at each cycle. Instead, distinct calibration data are acquired every Δ cycles. We determine a temperature estimate at each cycle with calibration data acquired before or at the cycle of interest. For the case where the power of the unknown is measured at time *t*, but calibration sources are not, we set ${\tilde{\theta }}_{t}={\tilde{\theta }}_{t^{*}}$, where *t*^∗^ *< t* is the calibration data acquisition time nearest to *t* that also precedes *t*. In the variogram plots (see Fig. 8), *τ* corresponds to the lag between the two cycles where temperature estimates are determined. The assumed calibration data for the two cycles may be the same or vary and may not be acquired at either cycle. Hence, interpretation of the variogram is more complicated than interpretation of the SVC metric.

For lags less than approximately 300 cycles, the variogram corresponding to Δ =500 cycles is consistently lower than the variograms corresponding to Δ = 1 cycle and Δ = 50 cycles (see Fig. 8). That is, according to the variogram, a less-frequent calibration scheme provides more stable temperature estimates relative to a more frequent calibration scheme for lags less than 300 cycles. This strange behavior is consistent with our claim that interpretation of variogram results is more complicated than interpretation of SVC results (for the examples studied here). Since SVC and the variogram are conceptually different, we do not expect the two metrics to agree.

### C. Other Calibration Schemes

In this article, we quantify stability for a calibration scheme where calibration data are acquired before the power of the unknown source is measured. Our stability metrics depend on both gain variations and possible temporal variations in the brightness temperatures of the reference sources. For the experimental data analyzed here, such brightness temperature variations affect our stability metrics in a very small or negligible way. In actual satellite systems, unknown source temperatures are typically determined with more complicated calibration schemes. For instance, the temperature estimate of the unknown source may depend on calibration data acquired before and after the time at which the power of the unknown source is measured. Next, we discuss a possible modification of our primary stability metric for application to more complicated calibration schemes.

Suppose that we have fine-scale calibration data at every time *t*_*u*_ where the power of the unknown source is measured. Furthermore, suppose that we subsample these data to produce data on a coarse temporal grid, where the interval between successive samples is Δ. Suppose that the closest time to *t*_*u*_ where there is coarse-scale calibration data is *t*_∗_ = *t*_*u*_ + *τ*. Denote the temperature estimate of the unknown source at *t*_*u*_ determined with the closest coarse-scale calibration data based on the more complicated, but unspecified, calibration scheme as

(20) $$\tilde{T}\left(t_{u},\tau \right)$$

and define a residual

(21) $$\epsilon \left(t_{u},\tau \right)=\tilde{T}\left(t_{u},0\right)-\tilde{T}\left(t_{u},\tau \right)$$

where $\tilde{T}\left(t_{u},0\right)$ is determined, in part, from fine-scale calibration data. Assuming that the mean square value of *ϵ*(*t*_*u*_, *τ*) exists and does not depend on *t*_*u*_, a natural candidate for a modified version of SVC is

(22) $${\mathrm{SVC}}_{\text{extend}}(\tau )=\sqrt{\frac{1}{2}<\epsilon^{2}\left(t_{u},\tau \right)>}$$

where the expectation would be over all possible values of *t*_*u*_ where −Δ*/*2 ≤ *τ* ≤ Δ*/*2. One would estimate SVC_extend_(*τ*) from experimental data in a way similar to how SVC is determined [see (7)]. The other steps to determine the component of uncertainty due to infrequent calibration would be similar to those listed at the end of Section IV-A. Application and analysis of this extended metric to more complicated calibration schemes is worthwhile but beyond the scope of this article.

## V. Summary

We directly quantified the effect of infrequent calibration on the stability of microwave radiometer temperature measurements acquired with the NFRad and the MIR with a new metric. For the calibration scheme studied, we also identified a component of uncertainty in a temperature estimate at a particular time based on the lag between the times at which the unknown power and the powers of the calibration sources are acquired. In contrast, existing stability metrics such as the variogram and Allan variance do not provide an estimate of this uncertainty component.

We developed a nonstationary stochastic model for the NFRad and determined random uncertainties of an empirical prediction model of our metric by Monte Carlo simulation. For the NFRad, we demonstrated that warm-up effects produced discrepancies between robust and nonrobust implementations of our metric. Hence, we expect that discrepancies between the two implementations may facilitate identification of outliers. For comparison purposes, we also studied a secondary metric that quantified stability for the case where calibration data are acquired at a fixed time, but power measurements for the unknown source are acquired at variable later times.

For the NFRad and the MIR, we fit many candidate empirical prediction models to our observed metrics and selected a parsimonious model from those that agreed best with observed values according to a root-mean-square deviation criterion. Since the radiometer hardware are different and the temperatures of the reference sources and unknown sources are different, differences in the selected mathematical forms are not unexpected. In general, the mathematical form of the “best” empirical prediction model may vary for other radiometers.

In our analysis, we determine our metric from relatively short time series. For sufficiently long time series, one might determine metrics from contiguous time intervals and search for temporal variations in the expected value of the metrics. Given a sufficiently long time series, one might determine a metric for each of many contiguous time intervals and determine metric uncertainty due to random effects based on these repeat measurements.

Due to size and mass limitations, small satellites typically lack blackbody targets and utilize external targets for calibration. Because these external targets cannot be observed as frequently as internal references, our methods are relevant to small satellites. Our methods should be useful for ground-based calibration studies as well.

## Figures and Tables

**TABLE I:** Estimated Parameters and Their Associated Standard Errors for SVC and SFC Metrics for NFRad Data

Metric	Parameter	Estimate	Standard Error

*SVC*	*α*_1_	1.58	0.66
	*β*_1_	0.0231	0.65
	*γ*_1_	0.446	0.30

*SFC*	*α*_2_	4.74e-05	0.64
	*β*_2_	1.54	0.64
	*γ*_2_	0.0286	0.29

**TABLE II:** Estimated Parameters and Their Standard Errors for Empirical Models of SVC and SFC Metrics for the MIR Data

Metric	Parameter	Estimate	Standard Error

*SVC*	*a*_1_	1.51	NA
	*c*_1_	0.148	NA
	*e*_1_	−1.05e-05	NA
	*b*_1_	0.0957	NA
	*d*_1_	−7.70e-06	NA

*SFC*	*a*_2_	0.171	NA
	*c*_2_	0.0298	NA
	*e*_2_	3.88e-06	NA
	*b*_2_	0.0833	NA
	*d*_2_	−3.18e-06	NA

## Appendix

### Stochastic Model for NFRad data

We model the unobserved gain time series, *G*, as a stochastic process *G* =1 + *z*, where *z* is an ARIMA time series. Our discussion of ARIMA processes closely follows [34]. An ARIMA process is a stochastic process that can be “differenced” to yield a stationary and causal autoregressive moving average (ARMA) process. More specifically, if {*X*_*t*_} is an ARIMA(*p, d, q*) process, *Y*_*t*_ := (1 *− B*)^*d*^*X*_*t*_ is a causal ARMA(*p, q*) process, where *BX*_*j*_ := *X*_*j−*1_ and *d* is a nonnegative integer. If {*X*_*t*_} is an ARMA(*p, q*) process, it satisfies

(23) $$X_{t}-\phi_{1}X_{t-1}-\cdots -\phi_{p}X_{t-p}\;\;=Z_{t}+\theta_{1}Z_{t-1}+\cdots +\theta_{q}z_{t-q}$$

where {*Z*_*t*_} is white noise and *ϕ*_1_, …, *ϕ*_*p*_ and *θ*_1_, …, *θ*_*q*_ are model parameters to be determined. (In a causal ARMA model, *X*_*t*_ can be expressed as a weighted sum of realizations of the white noise terms at time *t* and all times earlier than *t*.) ARIMA processes where *d* = 0 are stationary ARMA(*p, q*) processes. The terms AR(*p*) and MA(*q*) are shorthand for ARMA(*p,* 0) and ARMA(0, *q*) processes, respectively.

A simple example of an ARIMA process is a Gaussian random walk (a Brownian motion sampled at uniformly spaced times) *X*_*t*_ = *Z*_1_ + *Z*_2_ + *· · ·Z*_*t*_, where *Z*_1_, *Z*_2_, …, *Z*_*t*_ are independent and identically distributed Gaussian random variables with mean 0 and variance *σ*^2^, and *X*_0_ = 0. Since the variance of *X*_*t*_ is *σ*^2^*t*, {*X*_*t*_} is not a stationary process. However, *Y*_*t*_ := (1 *− B*)*X*_*t*_ = *Z*_*t*_ is stationary with variance *σ*^2^ and mean 0. Thus, the Gaussian random walk is an ARIMA(0,1,0) process. Although nonstationary, one can define the generalized power spectrum of the Brownian motion process as *S*(*f*) *∝ f*^*−*2^ [35].

We stress that we simulate one realization of *G* that applies to all the sources as well as the observed *V*_off_ time series. In our analysis, we approximate *G* as a scaled version of *V*_off_. Based on this approximation, we determine an ARIMA model for *G* based directly from analysis of the observed *V*_off_ time series. In particular, we fit ARIMA models with varying orders to the *V*_off_ time series and select the model that minimizes the AICc [32], [33], with the auto.arima function [30] in R [31] (a public domain software system). This approach yields an ARIMA(2,1,3) model. Hence, our initial model for *z* is the following nonstationary time-series model:

(24) $$y_{t}=\phi_{1}y_{t-1}+\phi_{2}y_{t-2}+\theta_{1}e_{t-1}+\theta_{2}e_{t-2}+\theta_{3}e_{t-3}+e_{t}$$

where *y*_*t*_ = *z*_*t*_ *− z*_*t−*1_ and *y*_*t*_ is an ARMA(2,3) stochastic process. The *e*_*t*_ term is Gaussian white noise with variance $\sigma_{e}^{2}$.

**TABLE III:** Estimated Parameters for Initial Model Described by (24) and (25)

***β_off_* (V)**	2.632809
$\sigma_{e_{\mathrm{off}}}\;(V)$	2.851859e-05
${\hat{\phi }}_{1}$	1.9286789
${\hat{\phi }}_{2}$	−0.9554731
${\hat{\theta }}_{1}$	−2.2885013
${\hat{\theta }}_{2}$	1.8385697
${\hat{\theta }}_{3}$	−0.5402265
${\hat{\sigma }}_{e}(V)$	4.382406e-06

We do not expect a scaled version of the gain to exactly predict *V*_off_. Hence, we model the observed *V*_off_ times series as the product of the unobserved stochastic gain function *G* and a scale factor *β*_off_ plus additive Gaussian white noise, ${\tilde{e}}_{\text{off}}(t)$, as follows:

(25) $$V_{\text{off}}(t)=\beta_{\text{off}}G(t)+{\tilde{e}}_{\text{off}}(t).$$

We set *β*_off_ equal to the median value of the observed *V*_off_ time series. Estimation of the variance of the additive Gaussian white noise term, ${\tilde{e}}_{\text{off}}(t)$, in our model for *V*_off_ is nontrivial because *G*(*t*) contains additive white noise. We first estimate the variance of the additive noise in *V*_off_ at any time, $\sigma_{e_{\text{off}}}^{2}$, as the sample variance of the *V*_off_ time series. As a caveat, we expect that the sample variance of *V*_off_ is an inflated estimate of the additive noise variance for various reasons. Our goal was to get a conservative estimate of the additive noise variance and then estimate a scaled version (where the scaling factor is less than 1) of it in a later analysis. (The initial estimates of the variances of additive noise terms for each reference source were similarly obtained.) We estimate the standard deviation of the additive noise terms in our model [see (25)] for *V*_off_, ${\tilde{\sigma }}_{e_{\text{off}}}$, as

(26) $${\tilde{\sigma }}_{e_{\text{off}}}=\alpha \sigma_{e_{\text{off}}}$$

where *α* is an adjustable scaling factor. Later, we scale *e*_*t*_ in our (24) by replacing *e*_*t*_ with ${\tilde{e}}_{t}=\kappa e_{t}$ where *κ* is adjustable. However, in the initial model described by (24) and (25), we do not scale *e*_*t*_. Table III lists estimated model parameters for the model of *V*_off_ described by (24) and (25).

We model the voltage time series for any particular reference source as

(27) $$V_{\text{ref}}(t)=\beta_{\text{ref}}\;G(t)[1+\Theta (t)]+\alpha \;\epsilon_{\text{ref}}(t)$$

where Θ is an AR(1) time series. Thus, we have

(28) $$\Theta (t)=\gamma_{1}\;\Theta (t-1)+\delta (t)$$

where *δ*(*t*) is Gaussian white noise with adjustable variance $\sigma_{\delta }^{2}$ and *γ*_1_ falls in the interval (*−*1, 1). We stress that each simulated realization of the Θ time series applies to simulated time series that corresponds to the two reference sources and the unknown source. Like before, *β*_ref_ is a scale factor set to the median value of the reference time series of interest. The term *ϵ*_ref_(*t*) is Gaussian white noise, and *α* is the adjustable scaling factor discussed earlier. We list estimated model parameters determined from *V*_off_ and the reference sources directly in Tables III and IV. In Table V, we list parameters determined from a grid search, where we minimize the difference between the observed value of SVC and the mean value of 15 simulated realizations of SVC for all candidate values of the relevant model parameters.

**TABLE IV:** Estimated Model Parameters for Sources

	Ambient	Cryogenic	Warm
***β_ref_* (V)**	2.627375	2.628516	2.627342
$\sigma_{\epsilon_{\mathrm{ref}}}\;(V)$	3.037315e-05	3.042696e-05	3.071763e-05

**TABLE V:** Additional Model Parameters Determined by a Grid Search Where, for the Unknown Source, We Minimize the Mean-Squared Difference Between Observed SVC and the Mean Value of SVC Determined From 15 Sets of Simulated Time Series for the Two Reference Sources and the Unknown Source

*α*	*κ*	*γ*_1_	*σ_δ_*
0.2	0.25	0.985	3.2e-7

In our study, we assume that the expected value of observed and simulated metrics exist. As a technical aside, for our Gaussian noise model, the expected value and the expected squared value (and hence the theoretical variance) of our simulated metric may not exist because the possible values of simulated normal (Gaussian) random variables are unbounded. To make this discussion more clear, we note that if observed powers are realizations of independent Gaussian random variables, the expected value and variance of the temperature estimate [see (1)] is a ratio of correlated Gaussian random variables, and its expected value and variance do not exist [36]. However, as discussed in [36], if the denominator is simulated from a truncated Gaussian distribution that sets a lower bound on the realizations, the ratio has well-defined expected value and theoretical variance. One can set this lower bound so that the probability of simulating a realization below the threshold from the original (untruncated) distribution is negligible. Hence, for theoretical reasons, it is reasonable to consider a truncated normal distribution model for noise. For instance, simulate realizations in the range (*−kσ, kσ*), where *σ* is the standard deviation of the noise and *k* is, say, 10. For our problem, we expect that such a truncation yields metrics with well-defined theoretical expected values and theoretical variance. Therefore, from a conceptual perspective, we should regard our simulation model as sampling from such truncated distributions. We stress that the number of realizations that one would have to simulate to observe a realization that differs from 0 by more or less than ten standard deviations is so large; the probability of observing one is negligible.
